# G4-Tetra DNA Duplex Induce Lung Cancer Cell Apoptosis in A549 Cells

**DOI:** 10.1186/s11671-016-1652-x

**Published:** 2016-10-01

**Authors:** Xiaobo Xu, YiZhuo Zhao, Hu Lu, Cuiping Fu, Xiao Li, Liyan Jiang, Shanqun Li

**Affiliations:** 1 Department of Respiratory Medicine, Zhongshan Hospital, Fudan University, Shanghai, 200032 China; 2 Department of Respiratory Medicine, Shanghai Chest Hospital, Shanghai Jiao Tong University, Shanghai, 200030 China; 3 Department of Nuclear Medicine, Zhongshan Hospital, Fudan University, Shanghai, 200032 China; 4 Shanghai Institute of Medical Imaging, Shanghai, 200032 China

**Keywords:** DNA tetra, AS1411, Delivery, Lung cancer therapy

## Abstract

The specific DNA is typically impermeable to the plasma membrane due to its natural characters, but DNA tetra structures (DTNs) can be readily uptake by cells in the absence of transfection agents, providing a new strategy to deliver DNA drugs. In this research, the delivery efficiency of tetrahedral DNA nanostructures was measured on adenocarcinomic human alveolar basal epithelial (A549) cells via delivering AS1411 (G4). The DNA tetra-AS1411 complex was rapidly and abundantly uptake by A549 cells, and the induced apoptosis was enhanced. Furthermore, biodistribution in mouse proved the rapid clearance from non-targeted organs in vivo. This study improved the understanding of potential function in DNA-based drug delivery and proved that DTNs-AS1411 could be potentially useful for the treatment of lung cancer.

## Background

Lung cancer is the leading cancer-related death cause in the world [1]. Despite therapeutic advances, the accounting is increased every year. Medicine therapy is an important scheme in lung cancer treatment due to the complexity in lesions [2, 3]. By now, though traditional therapy and some new-developed therapy of lung cancer have been eutherapeutic, effective drug delivery is still absent.

A special molecular DNA, G-quadruplex (also known as G4 structure), is formed in nucleic acids, and the sequences are rich in guanine [4, 5]. The guanine tetrad is formed through Hoogsteen hydrogen bonding by every four guanine, and two or more guanine tetrads can stack on top of each other to form a completed G-quadruplex [6, 7]. AS1411 is a G-rich quadruplex oligodeoxynucleotide that binds specifically to nucleolin and has been proved as an active oligo-drug for tumor therapy. AS1411 has been used as an anti-tumor drug or tumor-targeting agent [8–11]. The previous studies has proved that AS1411 can be taken up in cancer cells by macropinocytosis (fluid phase endocytosis) and distributed in the cells subsequently [12, 13]. As a high effective aptamer, AS1411 can recognize and bind nucleolin, the target protein in both cytomembrane and nucleus [14]. Meanwhile, the expression of nucleolin, which induces tumor cells anti-apoptosis, is usually higher in tumor cells [15, 16]. Hence, AS1411 is considered a precise inhibitor for nucleolin and relative signal pathway. The therapeutic effect in phase I clinical trials of patients is obvious, indicating that AS1411 is well tolerated with much of cancer treatment. In further studies, AS1411 was proved as a significant apoptotic inducer in adenocarcinomic human alveolar basal epithelial (A549) cells even lung tumor tissues [17]. However, the intracellular delivery of naked AS1411 is hardly possible due to naked oligodeoxynucleotides are not through the cell membrane directly in common serum.

Nano-drug delivery system, especially DNA nanostructure-based carrier, has already been a promising approach to assist AS1411 into target cells. The emergence of nano-biotechnology has provided widely opportunities for exogenous naked nucleic acid delivery in target cells [18]. DNA nanostructures have been employed as an efficiency drug carrier into cells because of their suitable sizes, structures, and synthesis. In previous studies, immunostimulatory CpG oligonucleotides are delivered into target cells as a significant immune-stimulating drug by DNA tetrahedra structures (DTNs) [19, 20], a 3D molecular cage formed with four DNA chains according to the principle of Watson-Crick base pairing [21]. Compared with naked DNA, DTN-compacted nanostructures offer greater flexibility in design and functionality and probably higher stability in cells. This discovery advocates new opportunities for DNA-based bio-interaction and smart drug delivery into live cells.

**Fig. 1:**
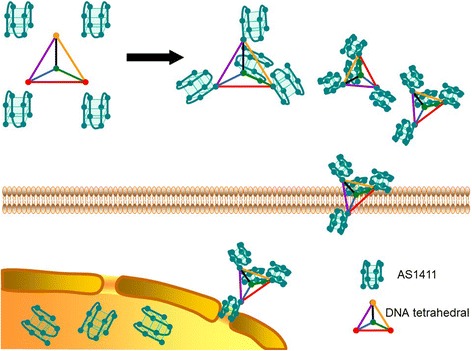
The mechanism of DTNs-AS1411 delivery system. AS1411 is linked with DTNs and then transported into A549 cells by DTNs. The main target site of AS1411 is in nucleus

Normally, cellular uptake of DNA typically relies on positively charged transfection agents, which are special agents together with the DNA. DNAs are allowed to traverse the negatively charged cellular membrane by this strategy. Interestingly, some DNA artificial nanostructures (such as DTNs) can be internalized by mammalian cells without the aid of transfection agents. In this study, we design a new complex of DTNs and AS1411 (Fig. 1), which is defined as the functional drug with the delivery cargo of DTNs by self-assemble technology. We herein explore the whole process of DTNs-AS1411 intracellular delivery and the apoptosis-inducing effects on the A549 cells. Furthermore, the metabolic pathway of DTNs-AS1411 in mouse is examined via SPECT/CT technology.

## Methods

### Chemicals and Materials

Phosphate-buffered saline (PBS, pH = 7.4) was purchased from Sangon Co, Ltd (China), 3-(4,5-dimethyl-2-thiazolyl)-2, 5-diphenyl-2-H-tetrazolium bromide (MTT), sodium dodecyl sulfate (SDS), polyacrylamide, bis-acrylamide, and TARMA were purchased from Sigma-Aldrich Co, Ltd (USA). Dulbecco’s modified Eagle’s medium (DMEM), fetal bovine serum (FBS), and penicillin-streptomycin solution were purchased from Invitrogen. Other chemicals were purchased from Sigma-Aldrich Co, Ltd. Anti-Bcl-2, anti-Bax, and anti-Caspase-3 proteins were purchased from Abcam Co. Ltd (Great Britain).

### Synthesis, Quantification, and Characterization of DNA Sequence

All DNA sequences were synthesized and purchased from TAKARA Co, Ltd (Japan). The oligonucleotides were used as AS1411: 5′-d (GGTGGTGGTGGTTGTGGTGGTGGTGG)-3′, and an inactive control oligonucleotide: 5′-dTTT (CCTCCTCCTCCTTCTCCTCCTCCTCC)-3′. The extra-TTT nucleotides in control oligonucleotides are the conjunction site with DTNs. The sequences of DTNs were as below:

A:

5′-ACATTCCTAAGTCTGAAACATTACAGCTTGCTACACGAGAAGAGCCGCCATAGTA-(AAA)-3′

B:

5′-TTCAGACTTAGGAATGTGCTTCCCACGTAGTGTCGTTTGTATTGGACCCTCGCAT-(AAA)-3′

C:

5′-TCAACTGCCTGGTGATAAAACGACACTACGTGGGAATCTACTATGGCGGCTCTTC-(AAA)-3′

D:

5′-TATCACCAGGCAGTTGACAGTGTAGCAAGCTGTAATAGATGCGAGGGTCCAATAC-(AAA)-3′

The sequence—(AAA)—is the bonding site with AS1411 in every chain if necessary.

The concentration of nucleic acids in solution can be readily calculated from the absorbance at 260 nm (A260) by UV spectrophotometry (Shimadzu Co, Ltd, Japan). One microliter of every sample was diluted with 499 μL double-distilled water and then examined for the relevant absorbance. The formula of calculating DNA concentration is as: concentration in μg/mL = A260 × weight/optical density (OD) × dilution factor. The dilution factor is defined by 1000/amount of suspended oligo added for the dilution.

For the synthesis of DTNs, every quantity DNA sequence was added into a reaction tube with the ratio 1:1. Then, the reaction system was heated to 95 °C for 2 min and then cooled to 4 °C for 30 s by PCR (Bio-Rad Co, Ltd., USA). All synthesized DNA structures were analyzed with agarose gel electrophoresis (1.2 %).

For the synthesis of AS1411, every quantity DNA sequence was added into a reaction tube at room temperature for 1 h. Then, the production is analyzed with agarose gel electrophoresis (1.2 %).

For the synthesis of DTNs-AS1411, the extra-TTT nucleotide chain of DTNs and AS1411 were the special sequences for synthesis. The reactions were heated to 95 °C for 2 min and then cooled to 4 °C for 30 s by PCR. The amount of AS1411 in each complex was controlled with the addition of nude AS1411 in the reaction system. All synthesized DNA structures were analyzed with agarose gel electrophoresis (1.2 %), and part of typical products was measured by dynamic light scattering.

### Cytotoxicity of DNA Structures

A549 cell lines were purchased from Cell Bank of Chinese Academy of Sciences (Shanghai). Then cells were cultured in DMEM medium with 10 % heat inactivated FBS, 0.15 % NaHCO_3_, 100 units/mL penicillin, 100 mg/mL streptomycin, and 2 mM L-glutamine at 37 °C in humidified air containing 5 % CO_2_.

Cytotoxicity was estimated using MTT assay. Firstly, cells were seeded in 96-well plates and cultured overnight to reach 80 % abundance. Then, the medium were removed and replaced with 100 μL fresh culture medium. Fresh media containing AS1411, DTNs, or DTNs-AS1411 at indicated concentrations were incubated with cells for 24 h. 20 μL 5 mg/mL MTT reagent solution was then added to each well, followed by 4 h incubation at 37 °C. Next, cells were lysed with 10 % acid SDS solution (pH = 2–3). After centrifugation, the absorbance of supernatant was measured at 570 nm by Microreader (Shimadzu Co, Ltd, Japan).

### Cellular Uptake of DNA Sequences

TAMRA is a stabled fluorescence with red emission (emission 542 nm, excitation 568 nm) in cell staining, which can be observed by confocal microscopy. In order to study the effects of A549 cells uptake ability, naked AS1411, DTNs, and DTNs-AS1411 at 100 nM were labeled with TAMRA (labeled in the 5′ of a chain of DTNs, such as AS1411). A549 were seeded on glass coverslips and incubated at 37 °C. After 24 h incubation, cells were washed with PBS and exposed to fresh culture medium containing different DNA sequences and then washed by PBS. The coverslips were mounted on a glass slide, and then the cells cultured on coverslips were fixed in 4 % paraformaldehyde for half an hour. After washing by PBS for several times, cells were then incubated with blocking buffer (2 % BSA) for 15 min. A special antibody (1:500) was then added and incubated overnight at 4 °C. After being washed, fluorescence-labeled secondary antibody was incubated for 1 h. Coverslips were then washed, and cells were observed using a laser confocal microscope (Leica TCS SP8, Germany).

### Cellular Apoptosis

In the present studies, DNTs were exploited as promising therapeutic agents due to their high solubility, design flexibility, and bio-safety. To test our hypothesis on apoptosis inducing, 3 × 10^5^ A549 cells were pretreated with 100 nM AS1411, DTNs, and DTNs-AS1411 independently for 6 h. Cell viability was then determined using MTT assays. Cellular apoptosis was characterized via western blot and TUNEL staining. A549 cells were plated and treated in six-well plates. After being treated with different DNA sequences, cells were washed several times with PBS, and harvested by SDS-loading sample buffer, and then boiled. Protein samples were then analyzed by 12 % SDS-PAGE and blotted to 0.22-μm diameter PVDF membranes. The samples were blocked for 30 min using 6 % BSA in PBST buffer (0.1 % Tween 20) and then incubated overnight at 4 °C with antibodies against Bcl-2, Bax, and active Caspase-3 (1:1000) independently. After being washed, the samples were probed with a goat anti-rabbit horseradish peroxidase-conjugated antibody (1:8000) for 1 h. The samples were then incubated with ECL plus and exposed to X-ray film. For TUNEL staining, the treated cell samples were treated with TUNEL staining kit following the instructions, and the corresponding samples were stained with DAPI to observe the amount of A549 cells.

### Biodistribution of DTNs-AS1411

Three Balb/C mice (8 weeks, 28.5 g in average) were purchased from Slac Laboratory animal (China) and recruited in this research. Animal care and all experimental procedures were performed under the approval of the Animal Care Committee of Fudan University. SPECT/CT imaging was operated in the Department of Nuclear Medicine, Shanghai Cancer Center, Fudan University. Preparation of ^99m^Tc-labeled DTNs-AS1411: Briefly, the mixtures of DTNs-AS1411 and NHS-MAG_3_ in HEPES buffer (pH = 8.0) were incubated at room temperature for 2 h and then purified on a P4 column with 0.1 M PBS as eluent. The peak fractions were collected and quantitated by UV spectrophotometry. For radiolabeling, 18.5 MBq ^99m^Tc-pertechnetate generator elute was added into a mixed solution of 40 μL (10 μg) of MAG_3_-DTNs-AS1411 in PBS, 15 μL tartrate buffer (pH = 8.5), and 5 μg SnCl_2_·2H_2_O in 0.01 M HCl. The solution was heated to 50 °C for 20 min. The ^99m^Tc-labeled DTNs-AS1411 was purified by size exclusion radio-HPLC. Aiming for the preliminary evaluation of biodistribution and in vivo metabolic mechanism, ^99m^Tc-DTNs-AS1411 (18.5 MBq/mouse) was administered to Balb/C mice via tail vein injection. At 3 h post injection, mice were anesthetized via 2 % isoflurane inhalation and scanned via NanoSPECT/CT (Bioscan, Washington, DC) for 20 min.

### Statistics

In this experiment, all significances were determined using Student’s *t* distribution (two-tailed; two-sample equal variance). *** equals *P* < 0.001; ** equals *P* < 0.01; * equals *P* < 0.05; ns means not significant.

## Results and Discussion

### Synthesis, Characterization, and Cytotoxicity of AS1411, DTNs, and DTNs-AS1411

**Fig. 2:**
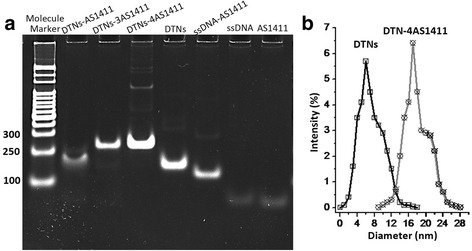
The characterization of AS1411, DTNs, and DTNs-AS1411. **a** The agarose gel electrophoresis of DNA nanostructures with details labeled on the band. **b** Diameters of DTNs and DTNs modified with 4 AS1411

1.2 % agarose gel electrophoresis was used to test the differences of molecular sizes among AS1411, DTNs, and DTNs-AS1411 due to that agarose gels are particularly suitable for separating molecular DNA of size and qualify range. The agarose gel electrophoresis (Fig. 2a) showed that DTNs-4AS1411 (4 AS1411 contained) was of the biggest molecular weight, and naked AS1411 (just 26 base pairs) was of the smallest molecular DNA size. Based on the molecular sizes reflected from the agarose gel electrophoresis, AS1411, DTNs, and DTNs-AS1411 formations were successfully synthesized. For the dynamic diameters of typical structures (Fig. 2b), the particle sizes corresponded to the molecule weight, and 8.2 ± 2.2 nm and 18.4 ± 3.7 nm were the particle sizes for DTNs and DTNs-4AS1411, respectively.

**Fig. 3:**
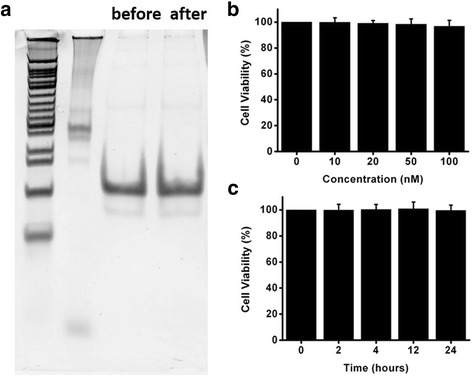
The stability of DTNs-AS1411 and cytotoxicity of DTNs. **a** The stability of DTNs-AS1411 in FBS (before and after incubation). **b** The viability of A549 cells with DTNs treatments at different concentrations. **c** The viability of A549 cells with DTN treatments at different time

Due to the basic character of DNA sequence, AS1411, DTNs, and DTNs-AS1411 can assemble automatically by base pair complementary mechanism at suitable temperature. The stability of DTNs-AS1411 measured via gel electrophoresis proved the stable performance against nuclease degradation in biological media, where the band intensity of DNA nanostructure remained as the same as before 4 h incubation (Fig. 3a).

For cytotoxicity, DTNs with different sequence and size were modified and confirmed safe to cells in previous studies. Cell compatibility of DTNs with different concentrations in A549 cells was measured via MTT assays (Fig. 3b, c). At concentrations between 10 and 100 nM, no significant effect on the viability of A549 cells was detected during the first 24 h, indicating that DTNs with 55 bp ssDNA are compatible to A549 cells.

### Cellular Uptake of DTN Structures

**Fig. 4:**
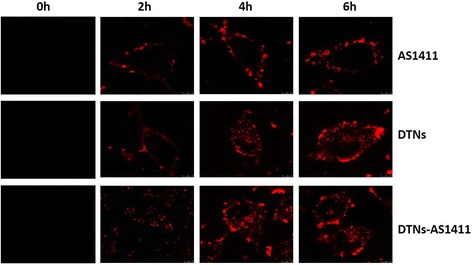
Cellular uptake of A549 cells with different DNA structures (*red* resulted from TRAMA)

The location and distribution of these DNA structures were examined in different times during the whole uptake process (Fig. 4). Significantly, a bright signal can be observed throughout A549 cells incubated with both DTNs and DTNs-AS1411. In contrast, there was minimal fluorescence in A549 cells with 100 nM AS1411 treated, suggesting that the ability of delivery by AS1411 itself was rarely. So, the presence of DTNs was crucial for efficient intracellular delivery. Besides, the fluorescence of DTNs obviously increased with the time. Both DTNs and DTNs-AS1411 were significantly higher than that of AS1411, indicating the high cellular uptake efficiency of DTNs. Furthermore, due to that DTNs and DTNs-AS1411 showed similar cellular uptake efficiency, the presence of DTNs motifs and the variation of the valence number had little influence on the efficiency of cellular uptake.

### Apoptosis Induced by Different DTNs in A549 Cells

**Fig. 5:**
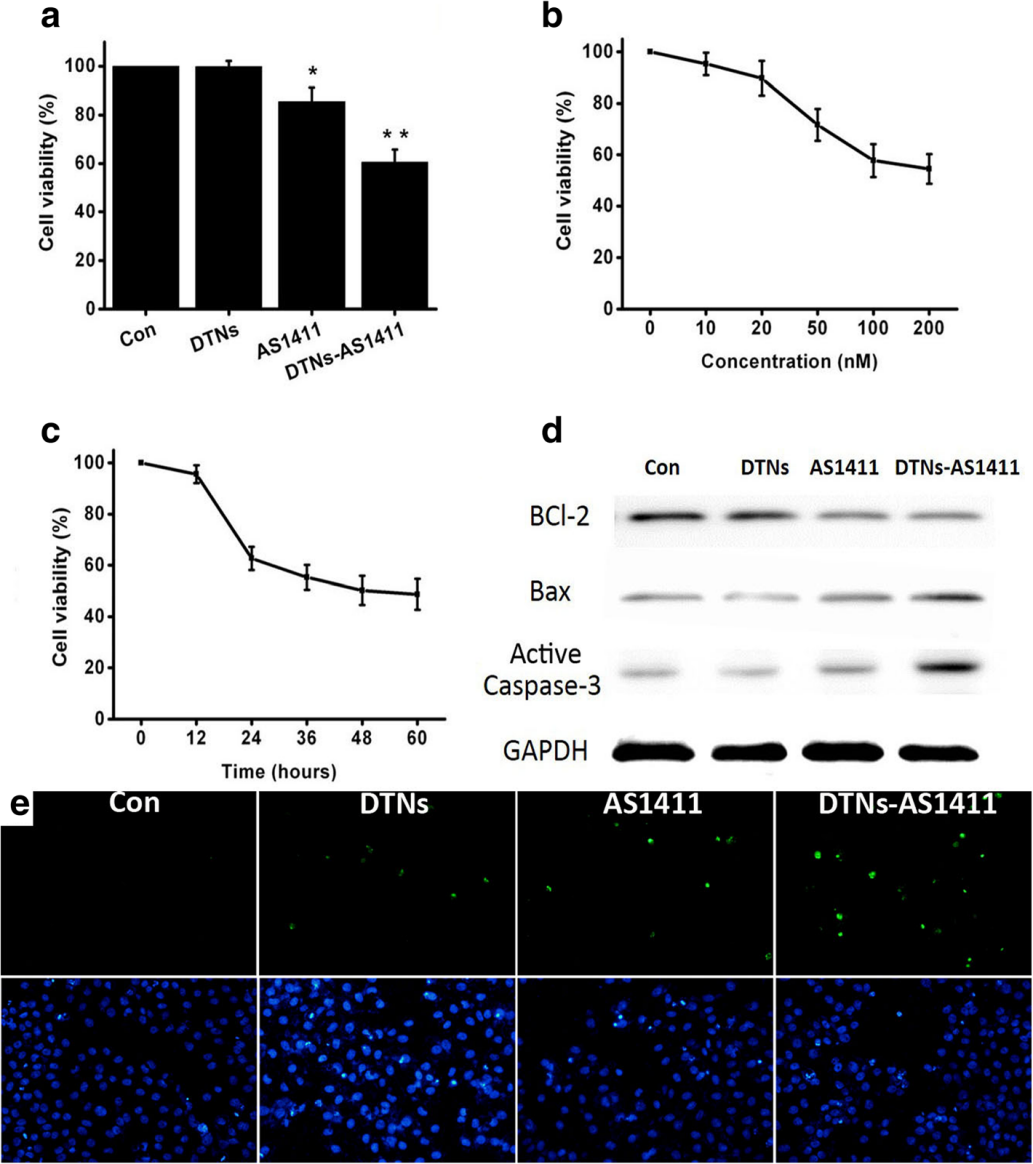
The apoptosis induced by the DNA structures in A549 cells. **a** The viability of A549 cells after being treated at 100 nm for 6 h. **b** The viability of A549 cells treated with different concentration of DTNs-AS1411. **c** The viability of A549 cells treated with different times of DTNs-AS1411. **d** The expression of BCl-2, Bax, and active Caspase-3 after different treatments. **e** TUNEL staining and DAPI staining of A549 cells treated with different drugs. **P value < 0.05, *P value < 0.1

There were no significant differences of viability between DTN treatment and control in A549 cells at 100 nM (Fig. 5a). The decrease of cellular viability induced by naked AS1411 was significant, but a more significant decrease was induced by the DTNs-AS1411 treatment. In Fig. 5b, c, the viabilities of A549 cells varied with the concentration of DTNs-AS1411 and the time length of co-incubation, indicating that AS1411 itself will not induce cytotoxicity obviously without the delivery of DTNs.

As an anti-apoptotic factor that expressed in normal state, BCl-2 has the ability to inhibit a wide variety of apoptotic signals. Reversely, Bax protein has the pro-apoptotic effect. Hence, the cellular Bcl-2/Bax ratio has been proposed as a key factor in the regulation of apoptosis in previous studies. Videlicet, Bcl-2/Bax ratio, makes cells resistant to apoptotic stage, while a low ratio induces cell death. Furthermore, Caspase proteinases, such as Caspase-3, make apoptotic signaling after aspartate residues, forming an activated protein in the apoptotic cell both by extrinsic and intrinsic pathways. For the western blot assays (Fig. 5d), a similar tendency to Fig. 5a was detected. The expression of BCl-2 decreased obviously in AS1411 and DNTs-AS1411 group. At the same time, the expression of Bax and active Caspase-3 increased. Yet, more significant changes were observed in DTNs-AS1411 group, indicating that the apoptotic-induction function of DTNs-AS1411 was more obvious. In the TUNEL staining results (Fig. 5e), DTNs-AS1411 lead to more apoptosis as well. Although DTNs are not the cytotoxicity factor, the delivery effect for AS1411 was proved. In addition, given that these nanostructures were stable because of their pairing, it is possible to carry AS1411 and other molecular guides into tumor cells for therapy.

### Biodistribution in Mouse

**Fig. 6:**
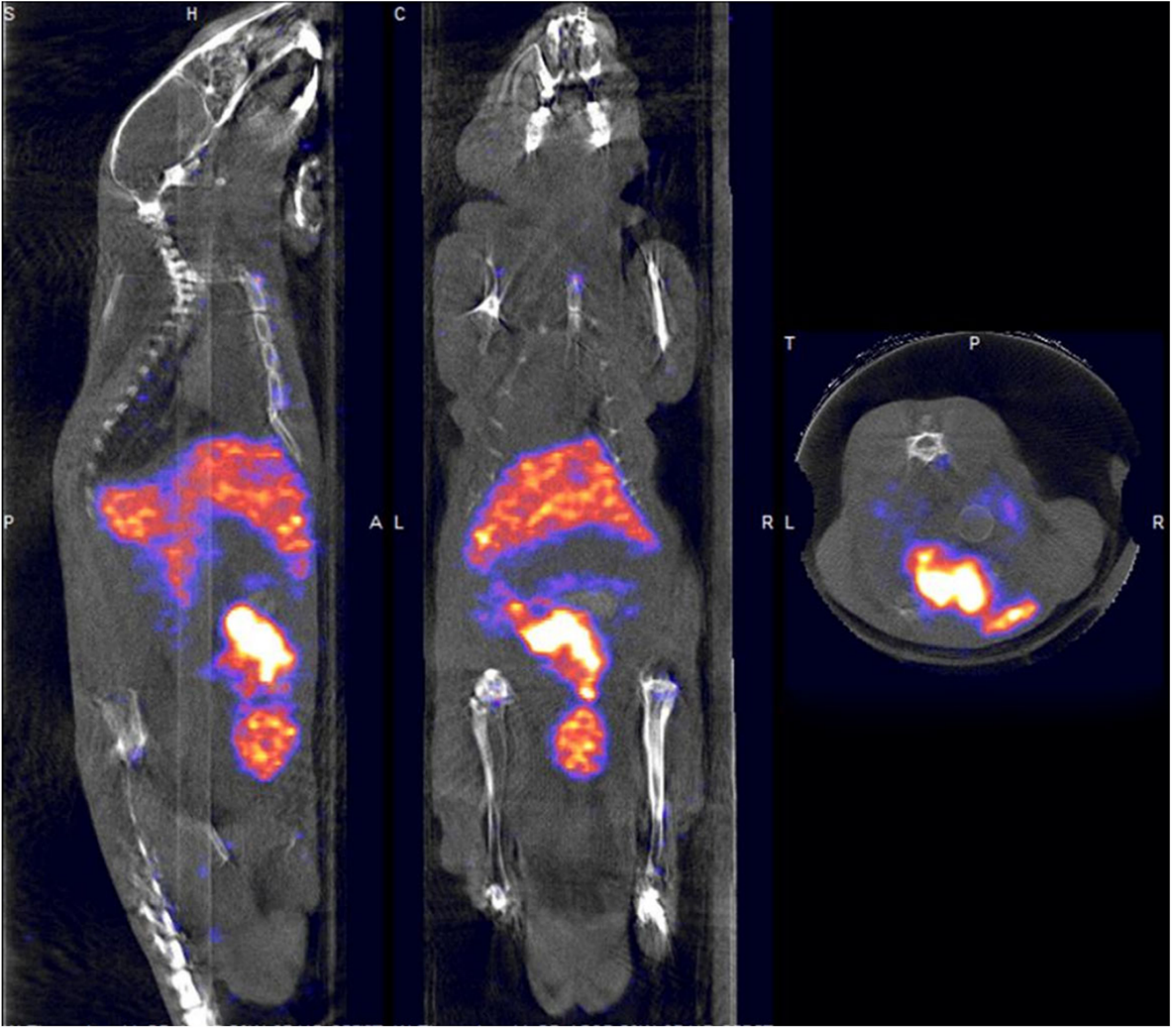
The biodistribution of ^99m^Tc-DTNs-AS1411 in Balb/C mice at 3 h post injection

Though naked AS1411 was proved as the actively anti-cancer in clinics, the in vivo metabolic process of DTNs-AS1411 is not clear. Hence, the biodistribution of DTNs-AS1411 was examined by SPECT/CT. After the intravenous injection of ^99m^Tc-DTNs-AS1411, no acute biotoxicity was observed during the first day post injection. Based on the SPECT/CT results (Fig. 6) scanned at 3 h post injection, more than 80 % DTNs-AS1411 have been expelled, indicating a rapid clearing rate in vivo, which is helpful to enhance the target/background ratio. For the normal mice, DTNs-AS1411 accumulated in RES-rich organs, such as the liver, and was mainly expelled via the bladder. Kidney metabolism was proved the major metabolic pattern of exogenous DNA structures in mice. This was a beneficial response to avoid the potential toxicity in vivo; due to that, DNAs did not obviously accumulate in non-target organs.

## Conclusions

DTN was confirmed as an excellent intracellular carrier for the transcellular transportation of AS1411, and DTNs-AS1411 is a potential strategy for lung cancer therapy with a low cost and high efficiency.
